# Plate fixation using parallelogram prism iliac bone grafts for clavicle oblique nonunion with shortening deformity: a case report

**DOI:** 10.1186/s12891-023-06468-w

**Published:** 2023-05-04

**Authors:** Ryogo Furuhata, Yuya Yokoyama, Atsushi Tanji, Shota Fujita

**Affiliations:** Department of Orthopaedic Surgery, Ashikaga Red Cross Hospital, 284-1 Yobe-cho, Ashikaga, 326-0843 Tochigi Japan

**Keywords:** Clavicle fracture, Nonunion, Oblique fracture, Scapular winging, Plate fixation, Bone graft

## Abstract

**Background:**

Plate fixation using a tricortical iliac bone graft can provide a high ratio of bone union and restore clavicle length in cases of atrophic nonunion. However, the surgical treatment of clavicle oblique nonunions with marked shortening deformity remains challenging and unresolved. Here, we describe a case of clavicle oblique nonunion with shortening that was treated using plate fixation with parallelogram prism iliac bone grafts.

**Case presentation:**

A 46-year-old man presented to our hospital with severe medial scapular pain. He had been diagnosed with a right clavicle mid-shaft fracture in a motorcycle accident 9 months earlier. He underwent conservative treatment, but radiographs and computed tomography showed clavicle oblique nonunion with marked shortening. Physical examination revealed no pain at the nonunion site; however, tenderness was noted on the medial side of the right scapula and protrusion of the inferior scapular angle was prominent. His symptoms interfered with daily life and required surgery. After release of the nonunion, we harvested the parallelogram prism bone grafts from the iliac crest. We inserted these bone grafts into the fracture ends and fixed them with lag screws, after which we performed plate fixation. Immediately after surgery, right medial scapular pain and scapula winging subsided. Bone union was achieved, and the length of the clavicle was restored at 9 months postoperatively.

**Conclusions:**

This case report provides new information on the surgical treatment of clavicle oblique nonunion with shortening deformity. The presence of medial scapular pain and winging scapula can be clinically problematic in cases of clavicle nonunion with marked shortening. Our case revealed that reliable bone union and clavicle length recovery can be achieved with plate fixation and iliac bone grafts.

## Background

Conservative treatment of clavicle mid-shaft fractures has provided satisfactory functional outcomes [1–4]; however, nonunion can occur in 5–15% of cases [5, 6]. Although clavicular nonunions are generally asymptomatic, surgery is indicated when symptoms such as pain at the nonunion site, limited range of shoulder motion, and shoulder muscle weakness interfere with daily life for a long period [7, 8].

To date, plate fixation with autogenous bone graft has achieved a high rate of bone union in atrophic nonunion of the clavicle [9–15]. In particular, in cases with marked clavicle shortening, tricortical bone grafts of the iliac crest have been used to restore the clavicle length [10, 12, 15]. For nonunions of transverse fractures with shortening, insertion of a rectangular bone graft into the clavicular defect and compression using plate screws provide firm fixation stability [7]. On the other hand, it is challenging to achieve good fixation of grafted bone in nonunions of oblique fractures; however, few reports are available on surgical techniques for such cases.

In this report, we describe a case of marked shortening of clavicle oblique nonunion. As a result of prolonged pain in the periscapular region and scapular winging, surgery was necessary in this case. This case suggested that plate fixation with parallelogram prism bone grafts of the iliac crest is beneficial for oblique nonunion of the clavicle with shortening.

## Case presentation

**Fig. 1:**
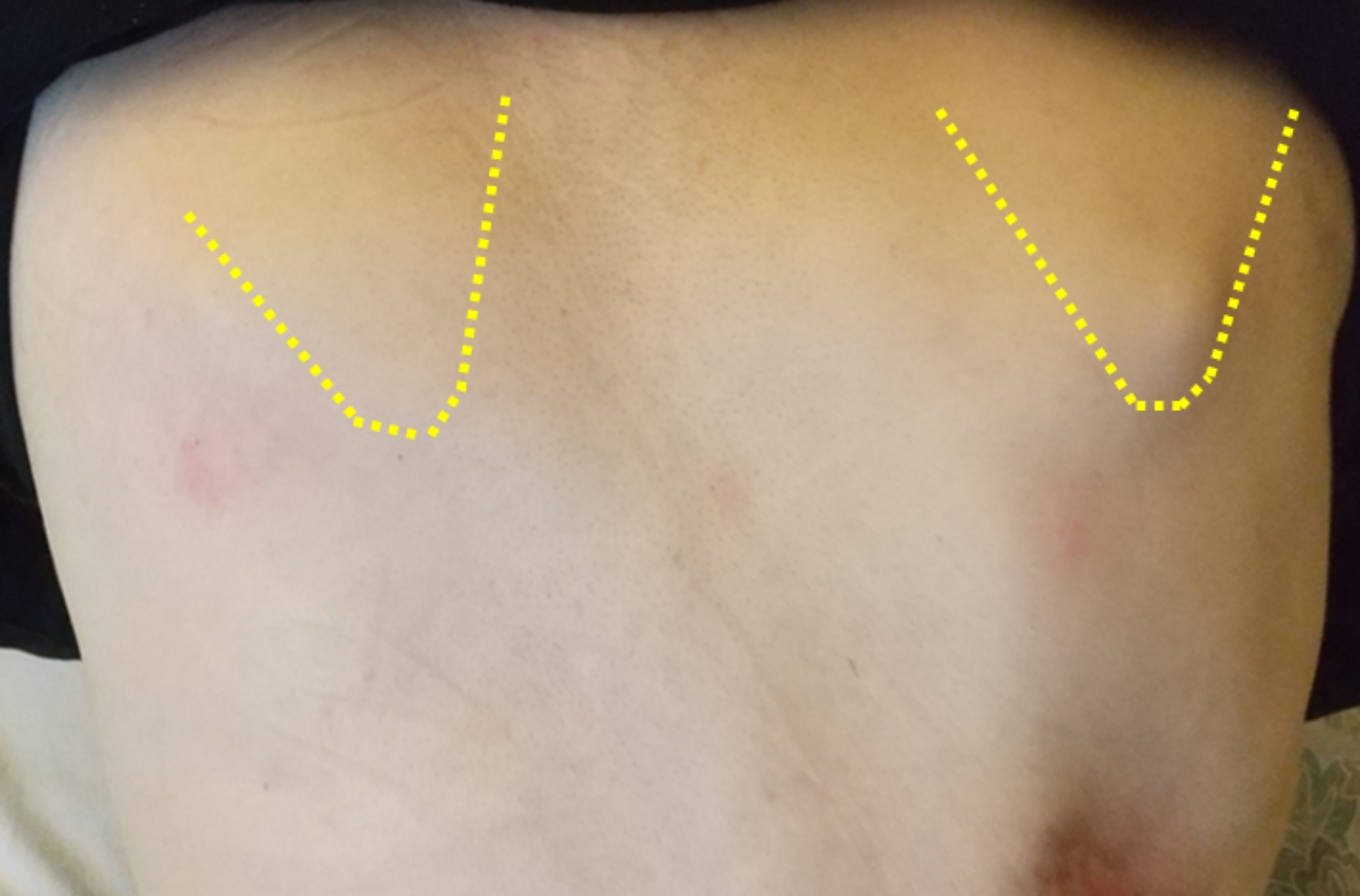
Appearance of the scapular. The superior angle of the right scapula was medial and inferior, and the inferior angle of the right scapula was inferior and protruded compared to the left scapular

**Fig. 2:**
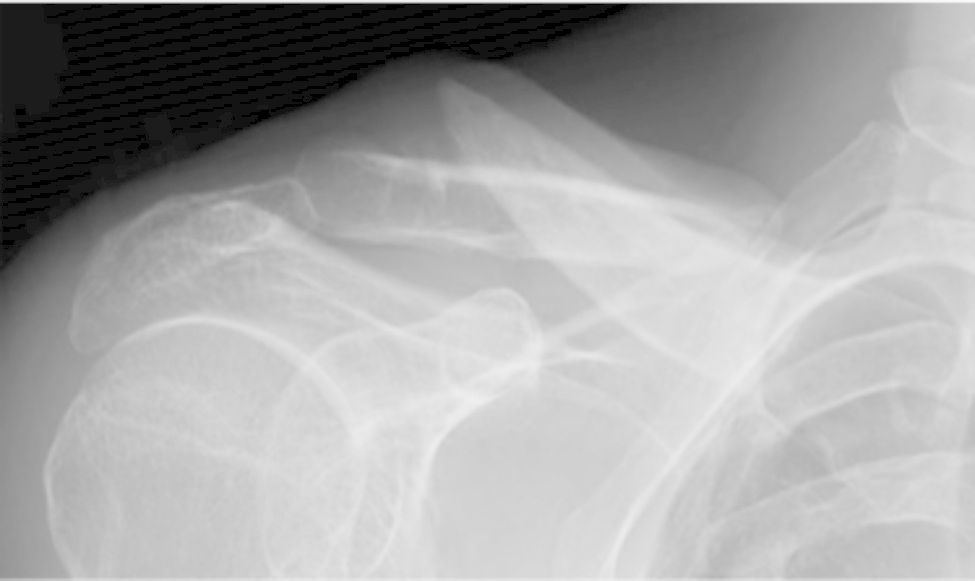
Preoperative radiographs showing atrophic nonunion with a large overlap of the fracture ends

**Fig. 3:**
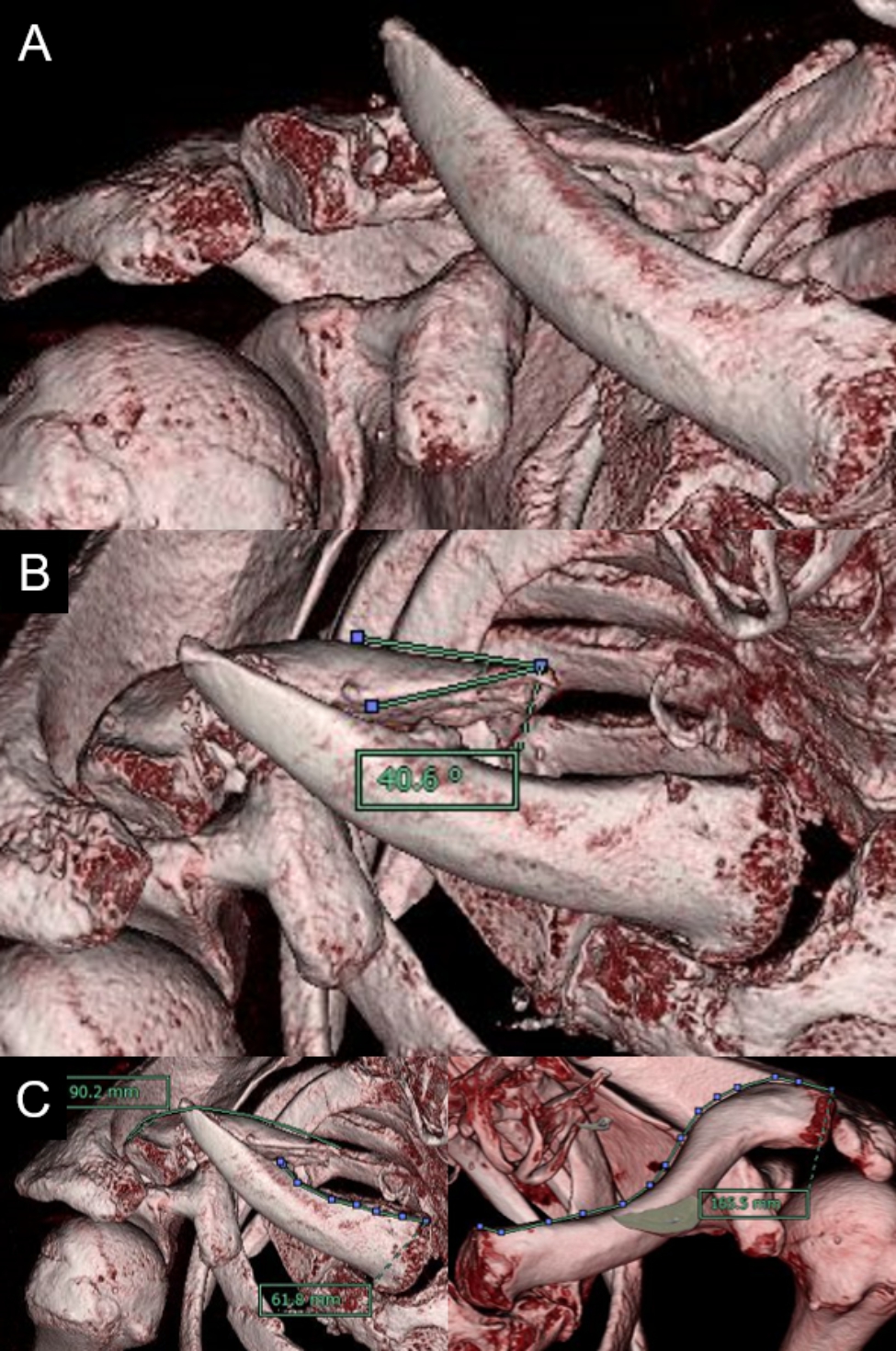
Computed tomography (CT) of the right clavicle 3D-CT demonstrated an atrophic nonunion of clavicle oblique fracture with 40.2° (A and B). The length of the distal and proximal fragments on the affected side was 90.2 mm and 61.8 mm, respectively, while the length of the clavicle on the unaffected side was 165.5 mm (C)

A 46-year-old man presented to our hospital with a complaint of medial scapular pain. He had been diagnosed with a right clavicle mid-shaft fracture and multiple rib fractures in a motorcycle accident 9 months earlier. He had been immobilized in a sling for 1 month and then underwent conservative treatment; however, X-ray radiographs indicated that bone union had not been achieved. He had a history of juvenile parkinsonism and had been taking ropinirole for 3 years. Physical examination revealed no pain at the nonunion site during shoulder motion; however, tenderness was noted on the medial side of the right scapula, and the pain worsened during anterior and lateral elevations of the shoulder joint. No gross atrophy of the shoulder girdle muscles was observed. The range of motion of the shoulder joint (right/left) was 110/140° for anterior elevation, and 0/15° for external rotation on the sides. The Constant score [16] was 37, and the visual analog scale (VAS) was 4.0 cm. Compared to the unaffected side, the superior angle of the scapula was medial and inferior, and there was marked protrusion of the inferior scapular angle (Fig. 1). There were no findings suggestive of vascular injury and electromyography demonstrated no evidence of nerve injury. Radiographs showed a right clavicle mid-shaft fracture with displacement (Fig. 2). Computed tomography (CT) showed no bone remodeling reaction at the fracture site (Fig. 3A). 3D CT measurements demonstrated that the fracture was an oblique fracture of 40.2° (Fig. 3B). The length of the distal and proximal fragments on the affected side was 90.2 mm and 61.8 mm, respectively, while the length of the clavicle on the unaffected side was 165.5 mm, indicating that the clavicle remained 13.5 mm shorter than that on the unaffected side following reduction (Fig. 3C). Based on these findings, he was diagnosed with oblique nonunion of the right clavicle with shortening. Since the patient had prolonged medial scapular pain that had interfered with daily life and work for a long period, we scheduled surgery.

**Fig. 4:**
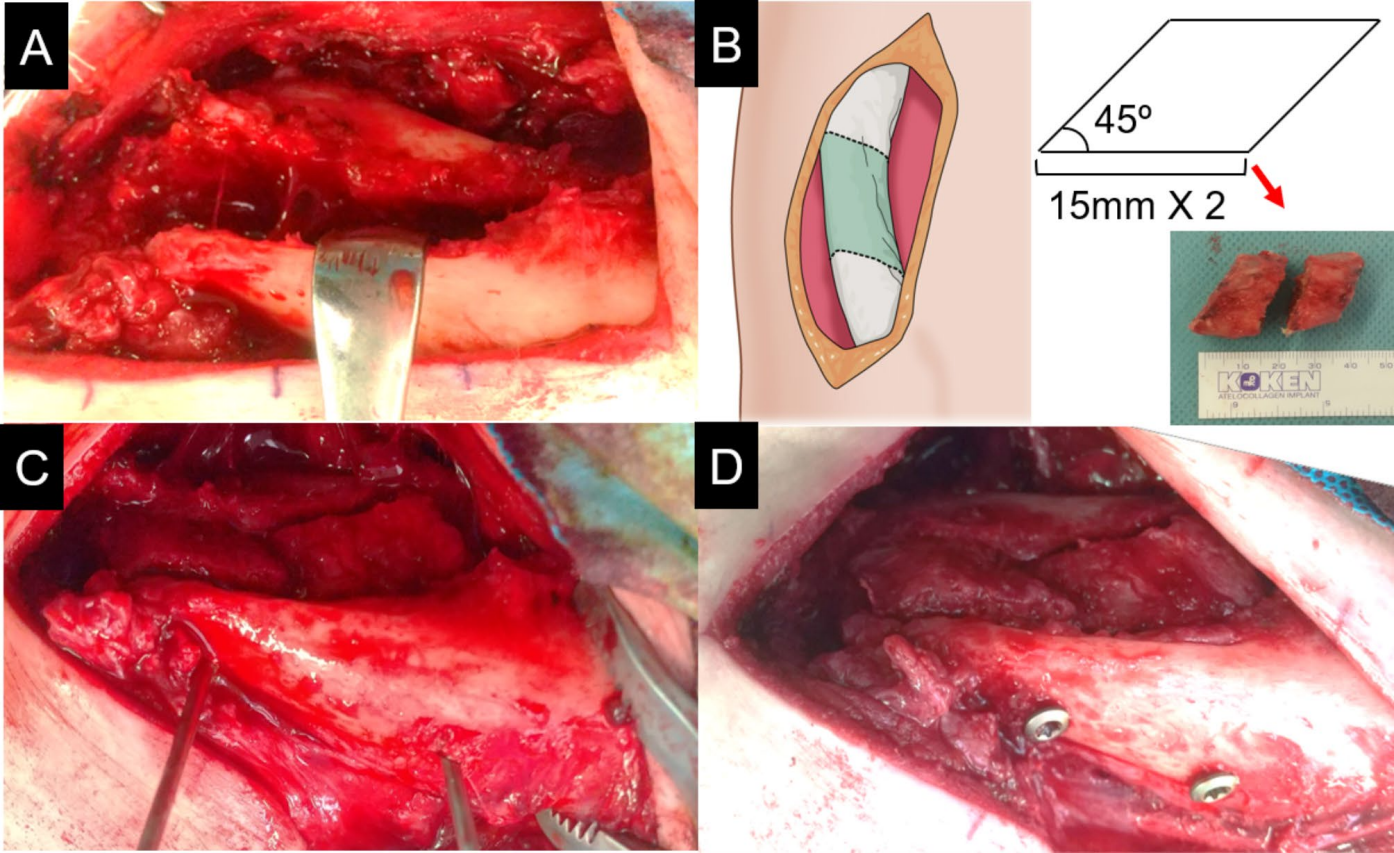
Intraoperative findings of the right clavicle. After releasing the nonunion, the fracture had a 1.5 cm gap between the fracture ends (A). We harvested two parallelogram prism bone fragments from the iliac crest according to preoperative measurement (B). After temporary fixation of central grafts with 2.0 mm Kirschner wires (C), we inserted two lag screws perpendicular to the fracture site (D)

**Fig. 5:**
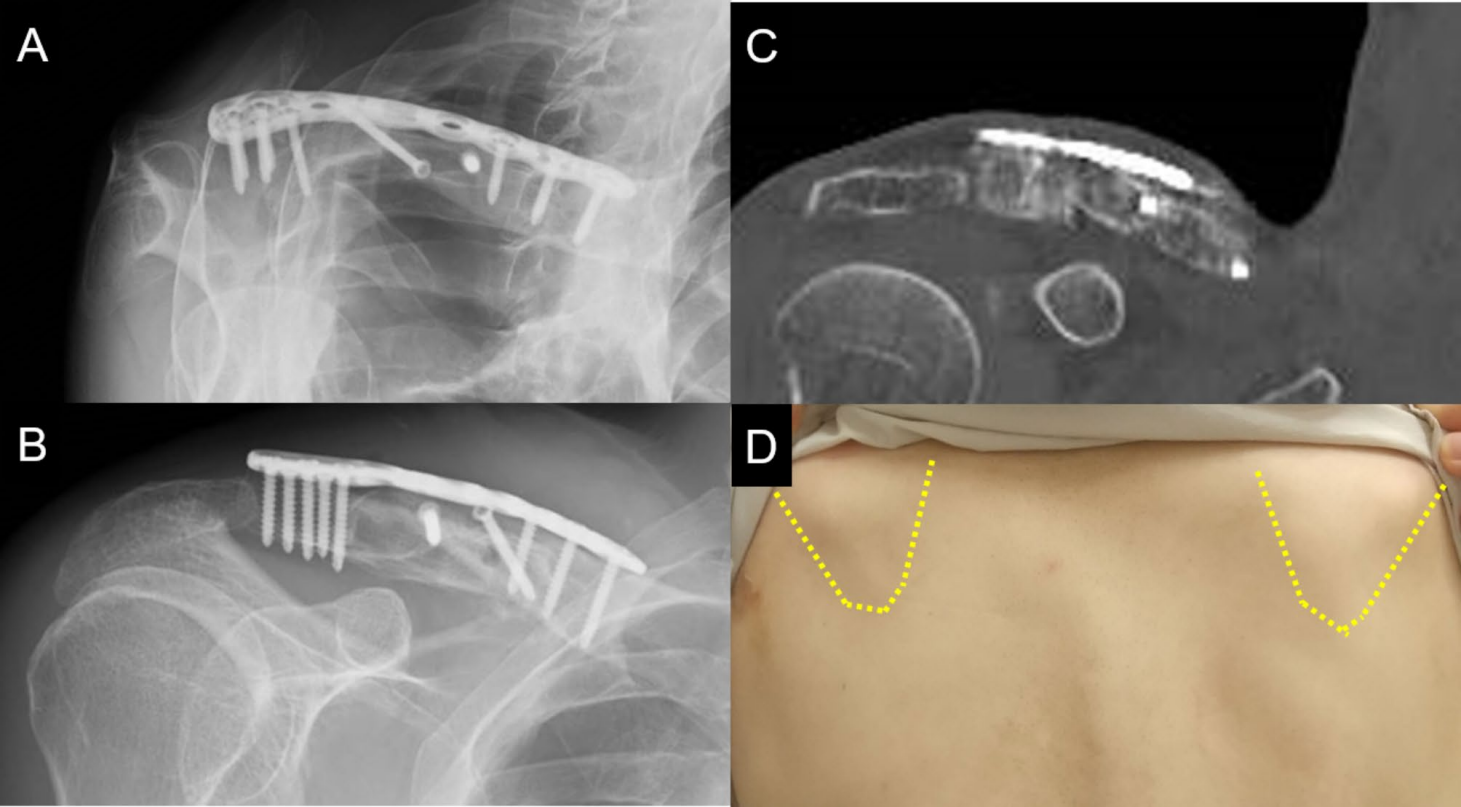
Postoperative imaging and physical findings. Radiographs of the right clavicle after plate fixation with parallelogram prism bone grafts (A and B). Computed tomography at 9 months after surgery showing bone union (C). Appearance of scapular showed that the medial and inferior translocation of superior angle of the scapula and protrusion of the inferior scapular angle subsided (D)

Under general anesthesia, surgery was performed in the beach chair position. A skin incision was made along the Langer’s line over the clavicle. A large amount of fibrous tissue was observed around the fracture site. We debrided the fibrous tissue and drilled the fracture ends to open up the intramedullary canal. There was a significant gap between both ends of the fracture (Fig. 4A); therefore, we performed iliac bone grafting. According to preoperative measurements, we planned to harvest a column iliac block with a base of 13.5 mm and an angle of 45°. We exposed the iliac crest, created a bony foramen in the iliac crest with a 2.0 mm Kirchner wire. We harvested two parallelogram prism bone fragments from the iliac crest with a chisel (Fig. 4B). The two iliac bone grafts were inserted into the clavicular gap side by side. After temporary fixation with 2.0 mm Kirschner wires through the proximal bone fragment, central bone graft, and distal bone fragment (Fig. 4C), we inserted two lag screws perpendicular to the fracture site (Fig. 4D). We applied Variax plate (Stryker, Kalamazoo, MI, USA) on the superior surface of the clavicle, and fixed the proximal and distal bone fragments (Fig. 5A and B). Postoperatively, the affected arm was kept in a sling for 3 weeks. From 1 week postoperatively, active range of shoulder motion training and rotator cuff strength exercises within ˂90° of elevation of the shoulder joint were commenced during rehabilitation. A full range of shoulder motion training was allowed 5 weeks postoperatively. Finally, scapular training was initiated 9 weeks postoperatively.

CT at 9 months postoperatively confirmed bone union (Fig. 5C). The clavicle lengths of the affected and unaffected sides were 163.8 and 164.9 mm, respectively, indicating recovery of clavicle length. The right medial scapular pain and scapula winging subsided (Fig. 5D), and the range of shoulder motion (right/left) was 130/140° for anterior elevation, and 10/15° for external rotation at 1 year postoperatively. The Constant score was 76, and the VAS was 0 cm at 1 year postoperatively.

## Discussion and conclusions

The present case indicated two clinical issues. First, this case demonstrates that clavicle nonunion with marked shortening deformity can cause persisted periscapular pain and scapular winging, which requires surgery. Although clavicular nonunions are usually asymptomatic, some cases present with symptoms such as pain at the site of the nonunion, shoulder fatigue, shoulder muscle weakness, and reduced range of shoulder motion, which can interfere with the ability to work or participate in recreational activities [7, 8]. In this case, the patient had a mild reduction in shoulder motion, but prolonged periscapular pain was the main factor that interfered with his daily life. Clavicle nonunions and shortened clavicle malunions have been reported to lead to scapular dyskinesis [17]. Especially in shortened clavicle deformities, increased anterior scapular tilt and internal rotation of the scapula result in medial and inferior translation of the superior angle and inferior translation and protrusion of the inferior angle of the scapula [18–20], which can sometimes lead to winged scapula [20, 21]. The preoperative physical findings in this case showed that the superior angle of the scapula translates medially and inferiorly, and that the inferior angle protrudes, similar to the results of these previous studies [18–20]. Although clinical risk factor has not been clarified for developing scapular winging in patients with clavicle nonunions or malunions, there are reports of severe scapular winging in those with multiple rib fractures [21] and injury to the shoulder girdle [22]. Additionally, it has been reported that clavicular shortening of ≥ 10% may affect scapular kinematics [19]. In this case, the concomitant multiple rib fractures and marked shortening of ≥ 10% of the clavicle length may have contributed to the onset of scapular winging, as reported in these reports [19, 21]. Even though persistent periscapular pain and scapular winging are not typical symptoms of the clavicular nonunion, they can be problematic when the clavicle is shortened, as in this case. Therefore, it is important to examine the scapular position in patients with clavicle nonunions clinically.

**Fig. 6:**
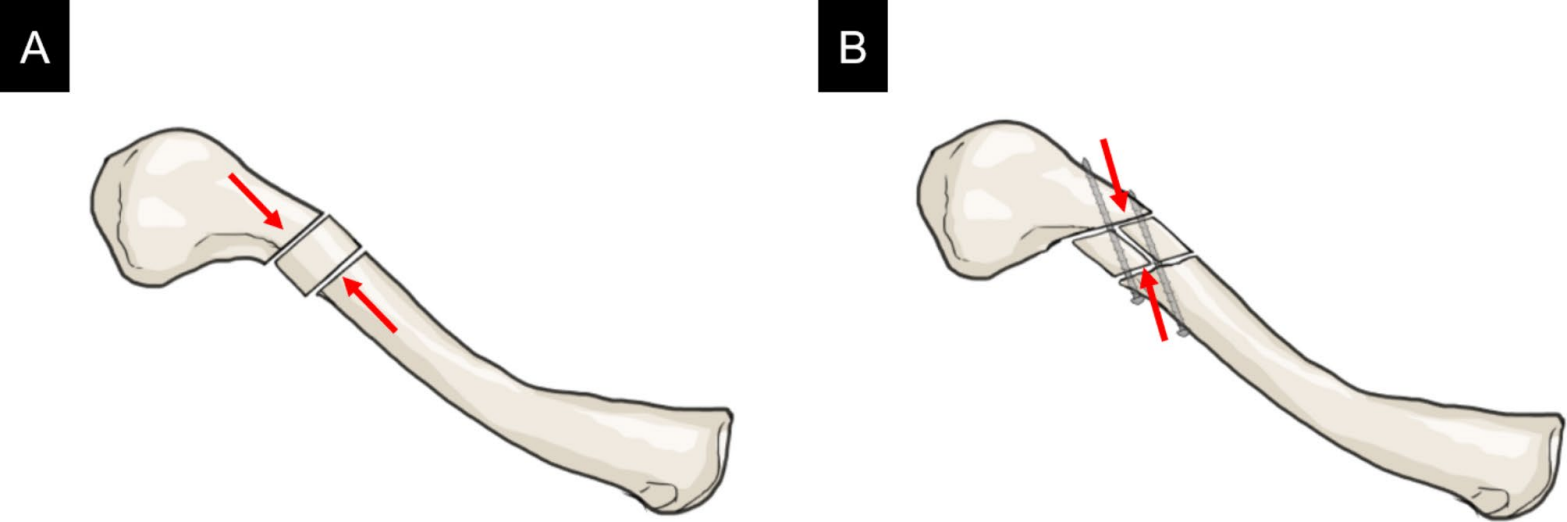
Schematic illustration of the surgery using iliac bone graft for atrophic nonunions with clavicle shortening. For nonunion of transverse fracture, insertion of the rectangular iliac bone graft into the fracture site and compression using eccentric drilling of screw holes (arrows) enhances the stability of fixation (A). For nonunion oblique fractures, parallelogram prism bone grafts increase the contacting surfaces of the limited grafted bone, and insertion of vertical lag screwed into the fracture site allows for rigid fixation of the central bone graft (arrows) (B)

Second, this case suggests that parallelogram prism bone grafts from the iliac crest are beneficial in treating clavicle oblique nonunions with shortening. Even though the use of autologous bone grafts are controversial in clavicle nonunion surgery, several studies have shown that plate fixation combined with a tricortical bone graft harvested from the iliac crest results in a high ratio of bone union and clavicle length restoration in atrophic nonunions with shortening of the clavicle [10, 12, 15]. For nonunions of transverse fractures, insertion of the rectangular iliac bone graft into the fracture site and compression using eccentric drilling of screw holes are thought to enhance the stability of fixation [7] (Fig. 6A). However, for oblique nonunions, the rectangular bone graft not only make it difficult to provide sufficient contacting surfaces with the fracture ends, but may also be subjected to forces that promote dislocation when compression is applied in the long axis direction of the clavicle. To combat these problems associated to oblique nonunions, parallelogram prism bone grafts were harvested to increase the contacting surfaces of the limited grafted bone and vertical lag screws were inserted at the fracture site to ensure rigid fixation of the central bone graft and the proximal or distal fragments (Fig. 6B). Another advantage of this procedure is that the parallelogram of the iliac graft can be adjusted according to the angle of the oblique fracture to maintain continuity in the clavicle cortex. Regarding the oblique bone grafts, such as the one used in this case, a study has used trapezoid iliac bone grafts in corrective osteotomies for malunited distal radial fractures [23]; however, no literature exists on surgery for nonunion. In our patient, reliable bone union was achieved postoperatively and the clavicle length was confirmed to recover to the same extent as that of the unaffected side.

In summary, this case report provides new information on the surgical treatment of clavicle oblique nonunion with shortening deformity. In cases of clavicle nonunion with marked shortening, medial scapular pain and winging scapula can be clinically problematic. The results of this case study showed that parallelogram prism iliac bone grafts fixed to plates led to reliable bone union and clavicle length recovery.

## Data Availability

Data that support the findings of this study are available from the corresponding author on reasonable request.

## List of abbreviations

VAS: Visual analog scale
CT: Computed tomography
